# Computational blueprints for cell fate programming

**DOI:** 10.1016/j.stemcr.2026.102929

**Published:** 2026-05-28

**Authors:** Pengyi Yang

**Affiliations:** 1 Computational Systems Biology Unit, Children’s Medical Research Institute, Faculty of Medicine and Health, The University of Sydney, Westmead, NSW 2145, Australia; 2 School of Mathematics and Statistics, Faculty of Science, The University of Sydney, Camperdown, NSW 2006, Australia; 3 Sydney Precision Data Science Centre, The University of Sydney, Camperdown, NSW 2006, Australia; 4 Charles Perkins Centre, The University of Sydney, Camperdown, NSW 2006, Australia

**Keywords:** ---

## Abstract

Cell fate programming enables applications in disease modeling, drug discovery, and regenerative medicine. Foundational studies established differentiation protocols, but their scalability is constrained by combinatorial complexity. Computational methods enable cell annotation, network inference, trajectory analysis, and have been applied to prioritize transcription factors and small molecules for cell fate programming, although prospective adoption for protocol design remains uneven. Single-cell and spatial omics, perturbation screens, and deep learning expand predictive scope while introducing challenges in domain shift, interpretability, and reproducibility. Here, I synthesize these approaches as pragmatic computational blueprints embedded in an iterative design-test-learn pipeline for cell fate programming.

## Teaser

In this article, Yang reviews computational approaches for cell fate programming and synthesizes them into a design-test-learn framework linking regulatory inference, perturbation modeling, and validation. The perspective highlights how integrating single-cell, perturbation, and deep learning methods may enable more predictive and reproducible cell-fate engineering.

## Introduction

**Figure 1:**
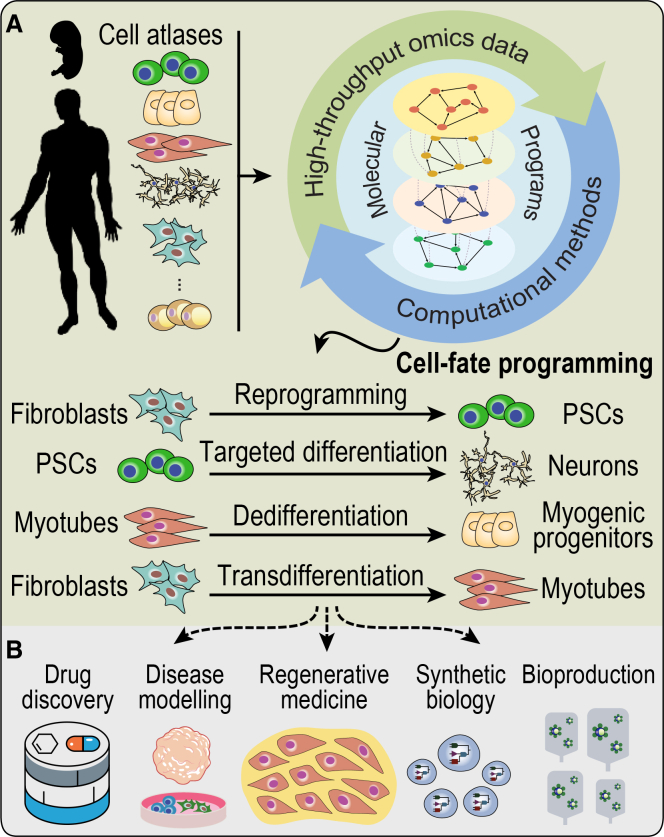
Schematic overview of cell fate programming (A) The computational methods and omics data that support its development. (B) Its implications across biomedical and biotechnological fields.

Cell identity and cell fate decisions are governed by molecular programs that integrate signaling, transcriptional, translational, and epigenetic regulation. Landmark discoveries demonstrated that these programs are malleable. Differentiated cells can be reprogrammed to a pluripotent state through forced expression of defined transcription factors (TFs) (Takahashi and Yamanaka, 2006), or directly converted into other cell types without passing through pluripotency (Vierbuchen et al., 2010). These breakthroughs established the conceptual and experimental foundations of cell fate programming (Lin et al., 2023) which encompasses four broad strategies (Figure 1A). In *reprogramming*, a differentiated cell is induced back to a pluripotent stem cell (PSC) state. In *directed differentiation*, a PSC or progenitor is guided toward a lineage of interest. In *dedifferentiation*, a mature cell is reverted to a progenitor-like state. In *transdifferentiation*, one differentiated cell type is converted into another without reverting to pluripotency. Across these strategies, the central objective is to modulate regulatory networks to generate desired cell types and states with high efficiency and fidelity, enabling applications in disease modeling, drug discovery, regenerative medicine, synthetic biology, and bioproduction (Morris and Daley, 2013; Srivastava and DeWitt, 2016; Tewary et al., 2018) (Figure 1B).

Early advances relied on empirical screening of TF combinations, culture conditions, and developmental cues. These efforts were essential in establishing feasible trajectories between cell states and generating the datasets that underpin current models. However, the combinatorial space of candidate regulators, dosages, temporal schedules, and microenvironmental cues expands rapidly and cannot be exhaustively explored experimentally. Computational approaches have therefore increasingly been used to characterize cell states, infer regulatory relationships, and prioritize candidate interventions (Cahan et al., 2021; MacArthur et al., 2009). In practice, these methods are most widely adopted for annotation, quality control, trajectory analysis, and post-hoc interpretation of cell identity, while their prospective use to design or optimize protocols remains comparatively limited.

Methodologically, the field has progressed in successive phases. Early theoretical work modeled gene regulatory networks (GRNs) as dynamical systems using Boolean and differential equation frameworks to explore multistability, oscillations, and stochastic transitions underlying fate decisions. With the emergence of bulk transcriptomics and epigenomics, network inference became increasingly data-driven, enabling systematic nomination of candidate regulators for reprogramming and differentiation (Cahan et al., 2014; Morris et al., 2014; Qin et al., 2020; Rackham et al., 2016; Xu et al., 2021). The advent of single-cell technologies marked a further shift. Large-scale atlases resolved discrete states and transient intermediates across tissues (Cao et al., 2020; The Tabula Sapiens Consortium, 2022), while multimodal and perturbation-based assays began linking cell states to regulatory mechanisms. These developments expanded the scope of GRN inference, enhancer-gene linking, trajectory reconstruction, and *in silico* perturbation analysis. Although such approaches have informed experimental hypothesis generation and in selected cases, protocol refinement, their reliability, and adoption vary across systems and applications. More recently, deep learning approaches have introduced new representational and predictive capabilities. Large pretrained models (“foundation” models) trained on millions of cells facilitate transfer learning for annotation and integration, and in some settings support perturbation prediction. Sequence-to-function models aim to connect *cis*-regulatory elements (CREs) to transcriptional outputs, while spatial and spatiotemporal frameworks relate cellular state to tissue context. These advances expand the computational toolkit available to protocol design for cell fate programming, yet they also raise challenges in interpretability, reproducibility, and domain shift between experimental systems.

While various reviews have summarized experimental and genomic advances in cellular reprogramming (MacArthur et al., 2009; Morris, 2026; Morris and Daley, 2013; Srivastava and DeWitt, 2016; Tewary et al., 2018), a comprehensive synthesis focused on how computational methods interface with experimental applications remains needed. Here, I examine how computational methodologies contribute to cell fate programming, distinguishing approaches that are routinely actionable today from those that remain promising but not yet reliable for prospective design. Foundations of computational cell fate modeling outlines the foundations, from dynamical systems to bulk-era network inference, highlighting both insights and limitations. The single-cell omics revolution covers the single-cell omics revolution. From identification to modulation moves from identification to modulation, discussing TF nomination and small molecule prediction, and their practical constraints in protocol optimization. Advances in deep learning for cell fate programming summarizes deep learning advances most relevant to cell fate programming. Computational evaluation and experimental validation of engineered cell fates considers evaluation and validation, focusing on standards required for computational predictions to meaningfully reduce experimental burden. Towards a design-test–learn pipeline for cell fate programming proposes a design-test-learn pipeline, linking computational prediction with experimental testing. Challenges and future directions discusses challenges and future directions toward predictive and reproducible cell fate programming, and (conclusions) provides concluding perspectives.

## Foundations of computational cell fate modeling

The foundations of computational cell fate modeling were established before the single-cell era, when regulatory processes were studied through mathematical abstractions and bulk population measurements. Two main approaches defined this stage. First, dynamical systems models provided conceptual frameworks for multistability, oscillations, and stochastic transitions underlying fate decisions. Second, bulk transcriptomic and epigenomic profiling enabled data-driven reconstruction of regulatory networks and nomination of TFs governing lineage specification. These early frameworks shaped modern thinking about cell identity, but were constrained by population averaging and limited context specificity, motivating the subsequent shift to single-cell and multimodal approaches.

### Mathematical modeling and dynamical systems

Classical computational approaches treated GRNs as dynamical systems in which stable attractor states corresponded to cell identities. Boolean network models (Glass and Kauffman, 1973; Kauffman, 1969) formalized switching behavior in regulatory circuits, while ordinary differential equation models demonstrated how feedback loops generate bistability and oscillations, exemplified by synthetic constructs such as the genetic toggle switch (Gardner et al., 2000) and repressilator (Elowitz and Leibler, 2000). Importantly, a series of experiments established stochasticity as a fundamental feature of gene regulation and single-cell measurements revealed intrinsic and extrinsic gene expression noise (Elowitz et al., 2002; Paulsson, 2004; Swain et al., 2002). These experimental findings have prompted the development of stochastic modeling frameworks that complemented deterministic descriptions. For example, probabilistic Boolean networks and related extensions introduced stochastic switching among regulatory rules, incorporating uncertainty and context dependence into discrete models (Shmulevich et al., 2002). Analytical treatments captured burst-like transcriptional dynamics (Shahrezaei and Swain, 2008), while potential landscape and probability flux formalisms provided global views of attractor stability and transition kinetics in nonequilibrium systems (Wang et al., 2008; 2011).

**Figure 2:**
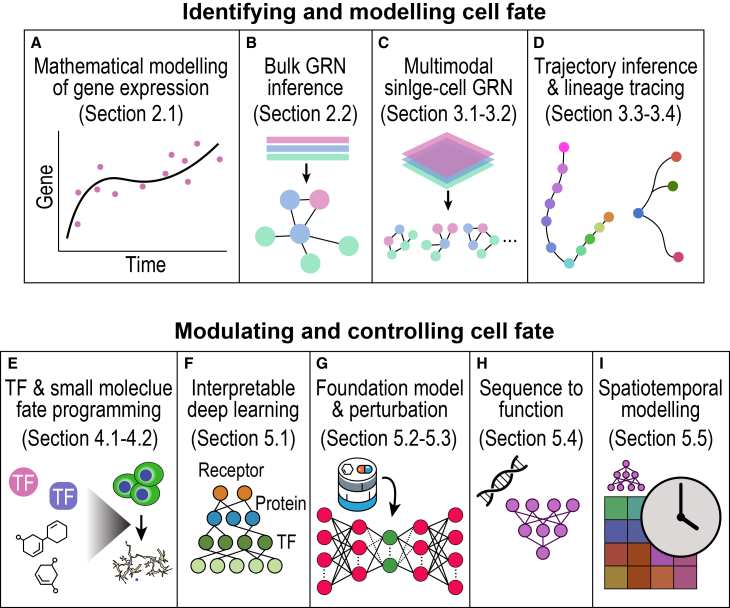
Schematic overview of computational methods for identifying and modeling cell fate and modulating and controlling cell fate (A) Mathematical and dynamical modeling of gene expression, (B) bulk GRN inference, (C) single-cell GRN inference and multimodal integration, and (D) trajectory inference and lineage tracing; and (E) TF and small molecule prediction, (F) interpretable deep learning, (G) foundation models and perturbation-aware approaches, (H) sequence-to-function modeling, and (I) spatiotemporal modeling.

Although these models are often limited to relatively small, well-characterized networks and require kinetic or time-series information for parameterization (Figure 2A), they remain valuable for their mechanistic interpretability. Concepts such as attractor stability, feedback-mediated multistability, and noise-driven transitions continue to underpin contemporary data-driven approaches to modeling and programming cell fate.

### Early network inference in the bulk omics

The proliferation of microarrays and bulk RNA sequencing enabled systematic reconstruction of GRNs from population-level data (Figure 2B). Early studies, such as the fibroblast serum response analysis by Iyer et al. (1999), demonstrated that co-expression patterns could reveal coordinated regulatory modules during dynamic transitions. Building on this principle, numerous frameworks formalized correlation- and regression-based network inference, including ARACNe (Margolin et al., 2006), GENIE3 (Huynh-Thu et al., 2010), Inferelator (Bonneau et al., 2006), CLR (Faith et al., 2007), PANDA (Glass et al., 2013), and DREM (Ernst et al., 2007), as well as motif enrichment tools such as iRegulon (Janky et al., 2014). These methods enabled regulator network analysis in bulk tissues.

Building on GRN inference, several frameworks explicitly linked network state to cell fate programming. Early approaches such as CellNet (Cahan et al., 2014) relied on bulk expression compendia to evaluate GRN status and prioritize TFs for fate conversion, while Mogrify (Rackham et al., 2016) leveraged promoter-level CAGE data and cell type ontologies to rank TFs mediating transitions between source and target cell identity. Recognizing the limitations of expression-only models, subsequent approaches incorporated regulatory priors such as sequence information, chromatin accessibility, and enhancer-gene linkage. Methods such as GarNet (Tuncbag et al., 2016), LISA (Qin et al., 2020), and ANANSE (Xu et al., 2021) combined transcriptomic and epigenomic data to infer context-specific regulators, while motif and sequence-based enrichment tools, such as DREME (Bailey, 2011), HOMER (Heinz et al., 2010), and KMAT (Guo et al., 2018), were widely used to nominate upstream TFs from gene sets. Comparative benchmarking studies indicate that methods incorporating priors such as chromatin accessibility information generally outperform expression-only approaches in recovering known reprogramming factors, although even the best-performing tools typically identify only a subset of experimentally validated regulators among top-ranked candidates (Hammelman et al., 2022).

Bulk omics approaches provided the first global views of regulatory architecture and represented an important step toward rational experimental design. However, population averaging obscures cellular heterogeneity, transient intermediates, and lineage bifurcations, while regulatory priors are often incomplete or biased toward well-studied TFs. These limitations motivated the transition to single-cell and multimodal technologies (the single-cell omics revolution), which enable network inference and perturbation analysis at cellular resolution for modeling cell fate.

## The single-cell omics revolution

Bulk-era approaches established regulatory logic but were constrained by population averaging. Single-cell technologies (Tang et al., 2009) mitigated this limitation by resolving cellular heterogeneity and enabling cell type- and state-specific modeling. When combined with multimodal profiling (e.g., scMultiome), they provide enhancer-gene linkages and TF-informed priors that strengthen regulatory inference (Figure 2C). These advances can be organized into four themes.

### Single-cell GRN inference

Single-cell GRN reconstruction often begins with identifying cell identity genes. While differential expression remains widely used (Soneson and Robinson, 2018; Squair et al., 2021), it prioritizes mean shifts rather than marker stability or specificity. Alternative strategies such as Cepo (Kim et al., 2021), COMET (Delaney et al., 2019), SCMarker (Wang et al., 2019), and scGeneFit (Dumitrascu et al., 2021) focus on stability, combinatorial discriminability, or geometric separability, yielding gene sets that reveal cell identity for downstream network reconstruction. Various single-cell GRN inference frameworks have been proposed, many of which integrate co-expression with regulatory priors. For example, SCENIC (Aibar et al., 2017) combines module inference with motif enrichment; PIDC (Chan et al., 2017) reduces indirect dependencies, and Inferelator 3.0 (Skok Gibbs et al., 2022) applies sparse regression with stability selection.

Together, these approaches enable state-resolved GRNs and candidate regulator nomination. However, sparsity, batch effects, and preprocessing variability limit reproducibility (Chen and Mar, 2018), and networks inferred from snapshot transcriptomes primarily reflect statistical associations rather than causal regulatory interactions. Their use in prospective protocol design therefore requires careful validation.

### Multimodal data integration

Multimodal single-cell assays address limitations of expression-only inference by jointly profiling complementary regulatory layers. Integration frameworks fall broadly into two categories, including latent space integration and regulatory linkage modeling. Latent integration methods such as Seurat’s weighted nearest neighbors (Hao et al., 2021), LIGER (Welch et al., 2019), MOFA+ (Argelaguet et al., 2020), GLUE (Cao and Gao, 2022), and deep generative models including totalVI and MultiVI (Ashuach et al., 2023; Gayoso et al., 2021) construct shared embeddings across modalities. These approaches facilitate joint clustering, annotation, and cross-modal alignment. Extensions such as Matilda (Liu et al., 2023) incorporate multi-task objectives to enhance biological signal extraction.

In comparison, regulatory linkage modeling approaches focus on enhancer-gene and TF-target linking. Co-accessibility tools such as Cicero (Pliner et al., 2018) and ArchR (Granja et al., 2021), enhancer prioritization models such as ABC (Fulco et al., 2019), and motif-based activity inference via chromVAR (Schep et al., 2017) refine regulatory hypotheses. SCENIC+ (Bravo González-Blas et al., 2023) and FigR (Kartha et al., 2022) integrate multimodal data to construct enhancer-centric regulons. Finally, CellOracle (Kamimoto et al., 2023) integrates chromatin accessibility and enables *in silico* TF perturbation analyses.

These methods increase biological plausibility by incorporating regulatory context, but do not by themselves establish causality. Outcomes remain sensitive to data quality, modality coverage, preprocessing decisions, and batch structure (Liu et al., 2025a), underscoring the need for benchmarking and validation.

### Trajectory inference and temporal dynamics

Single-cell GRN inference identifies regulators but does not reconstruct transition paths or directionality. Trajectory inference methods address this by arranging cells along putative developmental progressions (Figure 2D). Snapshot-based pseudotime approaches, such as Monocle (Trapnell et al., 2014), Diffusion pseudotime (DPT) (Haghverdi et al., 2016), Slingshot (Street et al., 2018), PAGA (Wolf et al., 2019), and Palantir (Setty et al., 2019), infer developmental ordering from manifold geometry. These methods estimate branch structure and differentiation potential but rely on cross-sectional assumptions.

Dynamic approaches sought to move beyond ordering alone. RNA velocity (La Manno et al., 2018) and scVelo (Bergen et al., 2020) estimate short-term transcriptional change, while optimal transport approaches, such as Waddington-OT, (Schiebinger et al., 2019) model couplings between time points. Recent extensions integrate spatial and multimodal information, including SpaTrack (Shen et al., 2025), Spateo (Qiu et al., 2024), and moscot (Klein et al., 2025).

Trajectory reconstruction is sensitive to manifold learning choices, root specification, sampling density, and modeling assumptions. Importantly, inferred trajectories from snapshot data do not establish lineage relationships or predict responses under perturbation. Their prospective utility therefore depends on complementary experimental validation.

### Lineage tracing

Trajectory inference (trajectory inference and temporal dynamics) arranges cells by state similarity to infer developmental progressions, but it does not record ancestry. Computational lineage tracing fills this gap by using heritable molecular barcodes to reconstruct clonal trees (Figure 2D), quantify fate biases, and validate regulator-driven hypotheses (Morris, 2026).

Tools such as Cassiopeia (Jones et al., 2020) and TiDeTree (Seidel and Stadler, 2022) infer lineage phylogenies from CRISPR-based recording systems. Integration methods including LineageOT (Forrow and Schiebinger, 2021) and CoSpar (Wang et al., 2022) combine lineage and transcriptomic information to improve directionality and estimate fate probabilities.

By linking ancestry with transcriptional trajectories, lineage tracing strengthens causal interpretation of GRN-nominated regulators. However, technical challenges including barcode dropout, editing inefficiency, sampling bias, and high experimental cost limit scalability. Lineage-resolved trajectories do not directly specify the molecular interventions required to redirect fate and must be integrated with perturbation data for prospective protocol design.

Foundations of computational cell fate modeling and the single-cell omics revolution collectively outline how methodological innovations have advanced the identification and modeling of cell identity and cell fate decisions. Table 1 summarizes representative methods, their key features, and typical applications, providing a reference point before we turn to strategies for actively modulating cell fate.

**Table 1:** Summary of representative methods underpinning the foundation of GRN inference and the single-cell revolution

Category	Methods	Key features	Applications
Mathematical modeling	Boolean networks; ODE models	Logical or continuous dynamics; attractor analysis; stochastic transitions	Conceptual models of cell-fate stability and switching
Bulk GRN inference	ARACNe; GENIE3; Inferelator; PANDA; iRegulon	Network reconstruction from bulk transcriptomics; correlation/regression-based inference; master regulator analysis	Identifying regulatory networks in bulk tissues
TF prioritization	CellNet; Mogrify; GarNet; LISA; DREME; HOMER	Sequence information, expression profile, and regulatory priors (enhancer-gene linkage, chromatin accessibility); ranks candidate TFs	Nomination of candidate regulators for reprogramming or differentiation
Single-cell GRN inference	SCENIC; PIDC; Inferelator	Cell-state-specific regulons; sparsity-aware inference	Modeling heterogeneity and dynamic transitions
Multimodal integration	Cicero; GLUE; MultiVI; FigR; Matilda; CellOracle	Joint RNA-ATAC integration; enhancer-TF-gene linking; shared latent embeddings	Multi-omic integration for GRN reconstruction
Trajectory and lineage tracing	Monocle; RNA velocity; Waddington-OT	Pseudotime ordering; dynamic transitions; lineage coupling	Developmental trajectories and reprogramming paths

## From identification to modulation

**Figure 3:**
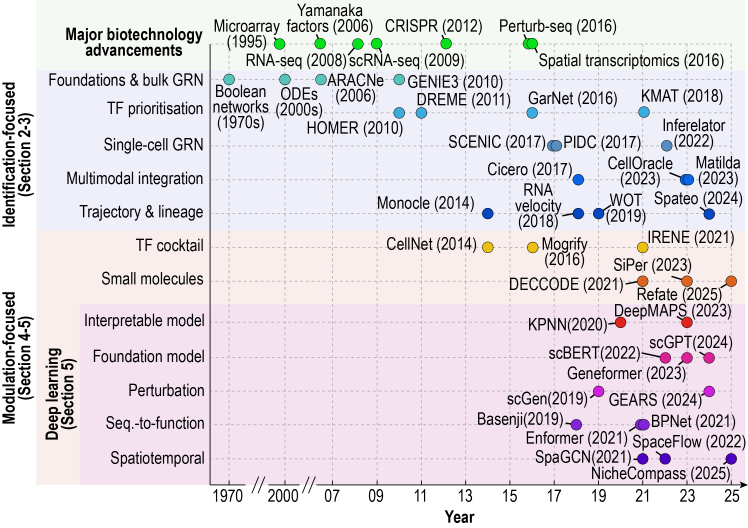
Timeline of represe Landmark computational approaches are organized chronologically and grouped by conceptual focus. Major biotechnological milestones are indicated to provide experimental context.

Computational approaches are increasingly applied not only to identify regulators and trajectories, but also to nominate interventions intended to modulate cell fate. These strategies prioritize TF cocktails or small molecules and explore dose, timing, and combinatorial parameters to enable cellular conversion. Although conceptually powerful, their prospective performance varies across systems, and most computational nominations require iterative experimental optimization. Figure 3 situates these approaches within the broader methodological progression from identification to intervention.

### Designing transcription factor cocktails

TF overexpression or suppression remains the most established strategy for modulating cell fate (Figure 2E). Computational design typically ranks candidate TFs that drive transitions between source and target states and produces shortlists for experimental testing. Early bulk-era frameworks, such as CellNet and Mogrify (Cahan et al., 2014; Rackham et al., 2016), reviewed in early network inference in the bulk omics, prioritized candidate TFs from large transcriptomic compendia, leading to experimental validation of regulators for cellular conversions (Morris et al., 2014). Integrative approaches incorporating chromatin accessibility, enhancer-gene linkage, and single-cell regulatory information the single-cell omics revolution have improved context specificity. Combinatorial design frameworks extend beyond individual factor ranking. Taiji-reprogram (Wang et al., 2021), TransSynW (Ribeiro et al., 2021), and IRENE (Jung et al., 2021) infer multi-TF panels based on network structure or enhancer-centric GRNs. Nevertheless, method performance remains dependent on the relevance of reference datasets and biological similarity between training and target systems. In practice, computational prioritization reduces search space rather than replacing experimental validation.

Recent studies increasingly couple computational nomination with systematic screening. Reprogram-Seq (Duan et al., 2019) quantified combinatorial TF effects using multiplex perturbation and single-cell readouts, while machine-guided approaches such as CellCartographer (Appleton et al., 2025) integrate modeling with iterative pooled screening in experiment-model feedback loops. Advances in perturbation infrastructure, including CRISPR-based transcriptional programming (Chavez et al., 2015), comprehensive TF libraries (Ng et al., 2021), and pooled perturbation profiling platforms (Kramme et al., 2021; Luginbühl et al., 2021), have expanded experimental throughput. However, the combinatorial explosion of TF sets and delivery configurations continues to limit exhaustive exploration.

A persistent challenge in TF-based programming is precise control of dosage and temporal dynamics. Although inducible systems, transient mRNA delivery, staged expression, and CRISPR-based modulation improve regulation, achieving appropriate stoichiometry and timing remains difficult. Suboptimal expression can yield off-target or heterogeneous states (Liu et al., 2025c), and engineered trajectories often diverge from developmental sequences. As a result, computationally prioritized TF sets typically function as experimentally testable hypotheses that require optimization to achieve robust conversion. In addition, dosage control depends not only on factor selection but also on expression-system design. Promoter choice, vector configuration, and delivery strategy influence expression levels, duration, and variability, and therefore form part of protocol design. Although emerging computational approaches may assist in designing regulatory elements with defined expression dynamics (see sequence-to-function modeling of regulatory elements), predictive construct design remains context-dependent and requires experimental calibration.

### Identifying small molecules for modulating cell fate

In parallel with TF-based strategies, small molecules provide a complementary route to steer cell fate by modulating signaling and gene expression (Figure 2E). Their reversibility and dose control make them well suited to staged differentiation protocols. Computational nomination generally matches source-target transcriptional contrasts against perturbational resources. Connectivity Map (Lamb et al., 2006) and its LINCS/L1000 expansion (Subramanian et al., 2017) established large-scale reference compendia, with tools such as L1000CDS2 (Duan et al., 2016) enabling signature-based prioritization.

Specialized tools adapt these principles for fate programming. DECCODE (Napolitano et al., 2021) identifies compounds facilitating direct conversions from transcriptional signatures; SiPer (Zheng et al., 2023) integrates scRNA-seq with curated perturbation resources to target signaling proteins; and Refate (Xiao et al., 2025) combines single-cell atlases with drug databases to prioritize compounds targeting PPIs and GRNs. These frameworks expand the design space beyond TF cocktails by incorporating signaling, epigenetic, and network-level priors. Nevertheless, their prospective performance depends strongly on the quality and relevance of reference atlases, the completeness of perturbation databases, and the extent to which drug responses generalize across cell types and culture conditions. Furthermore, many perturbational databases are derived from cancer or immortalized cell lines, which may not faithfully recapitulate developmental or differentiation contexts. As a result, predicted compounds often require substantial experimental validation, and transcriptional similarity does not necessarily translate into functional equivalence at the phenotypic level.

In practice, computational nomination improves efficiency in specific systems but rarely eliminates the need for empirical refinement. Most current approaches function as prioritization methods that reduce experimental search space rather than fully predictive design tools. To this end, strategies supporting an experiment-in-the-loop optimization paradigm have been established to iteratively refine protocol design (Del Vecchio et al., 2017; Kanda et al., 2022).

## Advances in deep learning for cell fate programming

Deep learning has introduced powerful representation-learning frameworks to single-cell and multi-omic analysis, capturing nonlinear structure in high-dimensional data. These approaches have improved annotation, integration, feature extraction, and perturbation modeling at scale. Applications span regulator identification and GRN refinement, transferable foundation-model embeddings, sequence-based regulatory modeling, and spatiotemporal integration of cellular states. Nevertheless, their predictive value for experimental cell fate programming depends strongly on the scale and relevance of training data, particularly perturbational datasets. The following sections outline recent advances while emphasizing their practical constraints for fate engineering.

### Interpretable deep learning for TF/gene discovery and GRN inference

Deep learning models with biologically structured latent spaces can assist in identifying regulators and coordinated gene programmes underlying cell identity. Compared with mean-shift differential expression, these approaches aim to capture higher-order patterns of gene activity. For example, siVAE quantifies each gene’s contribution to cell-type separation (Choi et al., 2023); scETM represents cells through topic-like gene programs (Zhao et al., 2021); and expiMap incorporates curated gene sets to produce pathway-level signatures (Lotfollahi et al., 2022). Benchmarking suggests that such models can complement traditional feature selection for robust identity gene discovery (Huang et al., 2023).

For GRN inference, interpretability is introduced either through model architecture or post-hoc explanation (Figure 2F). Knowledge-primed neural networks embed TF-target priors directly into network structure (Fortelny and Bock, 2020), while approaches such as scGeneRAI use relevance propagation to estimate TF-target importance (Keyl et al., 2023). Graph- and temporal-based extensions (e.g., DGRNS, STGRNS, and DeepMAPS) incorporate pseudotime, spatial, or multi-omic context to infer state-specific regulons (Ma et al., 2023; Xu et al., 2023; Zhao et al., 2022).

Despite improved interpretability, these models remain dependent on training data quality and regulatory priors. Inferred interactions typically represent statistical associations rather than validated causal regulation. Accordingly, they function primarily as hypothesis generating tools that require perturbational validation.

### Foundation models for single-cell data

Large pretrained “foundation” models have emerged as general-purpose representation learners for single-cell data (Figure 2G). Typically trained on millions of cells, these models treat genes as tokens and gene expression profiles as sequences, learning embeddings through objectives such as masked-token prediction. After pretraining, they can be fine-tuned for downstream tasks including annotation, batch correction, integration, perturbation modeling, and regulator prioritization.

Representative examples include scGPT (Cui et al., 2024), Geneformer (Theodoris et al., 2023), scBERT (Yang et al., 2022), and scFoundation (Hao et al., 2024), which demonstrate strong transfer performance for annotation, integration, and selected perturbation benchmarks when pretrained on large cell atlases. These models encode gene-gene and cell-state relationships within shared embeddings, enabling reference mapping, regulator prioritization, and exploratory counterfactual analyses in which cells are computationally shifted toward target states.

These models have clearly reshaped computational workflows for reference mapping and large-scale representation learning. However, their utility for experimental cell fate programming remains constrained by several factors, including corpus bias toward well-studied tissues, limited availability of large-scale perturbational training data, sensitivity to preprocessing and tokenization schemes, and domain shift across platforms or species. While foundation models provide scalable tools for representation and triage, robust protocol design for fate engineering still requires system-specific fine-tuning and experimental validation.

### Causal inference and perturbation modeling

CRISPR-based perturbation with single-cell readouts provide a crucial bridge between computational nomination and experimentally measured causal effects. Techniques such as Perturb-seq and CROP-seq combine CRISPRi-CRISPRn perturbations with scRNA-seq to profile hundreds to thousands of genetic perturbations in parallel (Adamson et al., 2016; Datlinger et al., 2017; Dixit et al., 2016). These datasets quantify how individual perturbations reshape transcriptional programs, regulons, and trajectory coordinates, supplying ground truth for computational model development and refinement.

Predictive modeling of lineage transitions further benefits from datasets in which interventions and differentiation trajectories are jointly observed. Large-scale TF and genetic screening studies that track cell state transitions at single-cell resolution in defined lineage contexts (Liu et al., 2025b; Parekh et al., 2018; Tsunemoto et al., 2018) provide such structured training examples. By systematically measuring how combinatorial regulators reshape differentiation paths, these datasets enable models to learn intervention-outcome relationships relevant to fate conversion. Relative to the vast combinatorial design space of TF sets, doses, and temporal schedules, available perturbation datasets remain sparse and unevenly distributed across tissues and developmental systems, limiting reliable generalization.

To address these limitations, several computational frameworks attempt to generalize beyond assayed perturbations. scGen transfers perturbation effects across cell types using a variational autoencoder (Lotfollahi et al., 2019); CPA disentangles basal state from treatment to extrapolate across dose, time, and drug combinations with uncertainty estimates (Lotfollahi et al., 2023); and GEARS integrates prior gene relationships to forecast outcomes of single- and multi-gene edits while identifying nonadditive interactions (Roohani et al., 2024). These models enable *in silico* triage of candidate interventions before experimental testing and can iteratively improve as new perturbation data are incorporated.

Nevertheless, reliable extrapolation to unseen combinations, higher-order TF sets, novel cell types, or long differentiation time courses remains challenging. Data sparsity, batch effects, cell cycle confounding, and domain shift can distort counterfactual predictions. Consequently, perturbation-aware modeling is most powerful when embedded within iterative experiment-model feedback loops, where predictions are prospectively tested and incorporated into subsequent training rounds. Expanding high-quality, lineage-specific perturbation datasets will be central to improving the reliability of computational blueprints for cell fate programming.

### Sequence-to-function modeling of regulatory elements

Deep sequence models aim to predict regulatory activity directly from DNA, offering a potential route toward *in silico* design of enhancers and promoters that modulate gene expression programs (Figure 2H). Convolutional architectures such as Basenji model long-range *cis*-regulatory dependencies to predict chromatin accessibility and transcriptional outputs (Kelley et al., 2018), while BPNet links motif grammar to TF binding at base-pair resolution (Avsec et al., 2021a). DeepSTARR enables quantitative modeling of enhancer strength from short sequences (De Almeida et al., 2022), and transformer-based models such as Enformer extend receptive fields to nearly 1 Mb to capture distal enhancer-promoter interactions (Avsec et al., 2021b).

Feature attribution approaches (e.g., DeepLIFT and saliency maps) highlight nucleotides predicted to influence regulatory activity, supporting prioritization of noncoding edits, enhancer CRISPRa-i targeting (Gilbert et al., 2013), or MPRA tiling experiments (Inoue and Ahituv, 2015). When integrated with enhancer-gene maps derived from multimodal data (multimodal data integration), these models link specific DNA elements to predicted transcriptional outcomes.

Despite impressive predictive performance on matched training contexts, sequence-to-function models remain sensitive to cell-type specificity, assay conditions, and training corpus composition. Models trained on *in vitro* datasets may generalize poorly *in vivo*, and predicted regulatory gains or losses require validation through perturbational assays. As such, these approaches currently function as design-support tools that prioritize candidate regulatory edits.

### Spatiotemporal modeling

Cell fate decisions are shaped not only by intrinsic regulatory programs but also by spatial organization, temporal dynamics, and microenvironmental cues (Figure 2I). With the expansion of spatial transcriptomics (Moses and Pachter, 2022), deep learning frameworks increasingly integrate single-cell states with tissue context to map where specific lineages emerge and which niche signals co-vary with commitment.

Methods such as Tangram (Biancalani et al., 2021), cell2location (Kleshchevnikov et al., 2022), and DestVI (Lopez et al., 2022) align or deconvolve single-cell profiles within spatial transcriptomic data. Graph-based approaches including SpaGCN (Hu et al., 2021) and NicheCompass (Birk et al., 2025) incorporate spatial adjacency and histological information to infer tissue domains and cell-cell communication. Latent embedding methods such as DeepST (Xu et al., 2022), stLearn (Pham et al., 2023), and SpaceFlow (Ren et al., 2022) integrate expression with spatial coordinates to model gradients and tissue organization.

These approaches extend cell fate modeling from isolated molecular states to tissue-level context, informing hypotheses about morphogen gradients, niche interactions, and temporal delivery of cues in organoid or co-culture systems. However, most current models remain descriptive, identifying spatial correlations rather than experimentally validated causal influences. Resolution constraints, sparsity, and experimental artifacts further limit predictive reliability. Consequently, spatiotemporal modeling currently serves primarily to contextualize fate decisions and explore environmental interventions. Sections From Identification to Modulation and Advances in Deep Learning for Cell Fate Programming focus on computational strategies for modulating and engineering cell fate, and Table 2 summarises representative methods, their key features, and typical applications.

**Table 2:** Summary of representative computational methods for modulation and deep learning in cell-fate programming

Category	Methods	Key features	Applications
TF cocktails	CellNet, Mogrify, IRENE	Ranks candidate TFs using transcriptomic and regulatory priors	Prioritizing TF sets; reducing combinatorial search space
Small molecules	DECCODE, SiPer, Refate	Matches transcriptional signatures to perturbational resources	Prioritizing candidate compounds for experimental testing
Interpretable models	KPNN, scGeneRAI, DeepMAPS	Biologically constrained architectures; attention or relevance attribution	Regulator prioritization; GRN refinement
Deep learning foundation models	scGPT, Geneformer, scBERT, scFoundation	Pretrained embeddings; transfer learning across datasets	Annotation, integration, perturbation triage
Perturbation modeling	scGen, CPA, GEARS	Learns perturbation-response patterns from CRISPR or TF screens	*In silico* prioritization of interventions; modeling combinatorial effects
Sequence-to-function	Basenji, BPNet, DeepSTARR, Enformer	Predict enhancer/promoter activity from DNA sequence	Regulatory element prioritization; guide design of edits
Spatiotemporal modeling	SpaGCN, SpaceFlow, NicheCompass	Integrates transcriptomics with spatial context	Contextualizing fate decisions; mapping niche influences

## Computational evaluation and experimental validation of engineered cell fates

Designing protocols for programming cell fate requires rigorous evaluation of both computational predictions and experimental outcomes. Computational metrics assess transcriptional fidelity, regulatory coherence, and trajectory consistency, while experimental assays validate identity, function, and safety. Beyond these, evaluation should determine whether computational models reduce experimental search space, generalize across datasets, and prospectively improve protocol design. This section discusses complementary approaches across identity scoring, regulatory and trajectory benchmarking, reference selection, and experimental validation.

### Computational identity scoring and fidelity assessment

Assessing whether engineered cells recapitulate their *in vivo* counterparts is central to evaluating cell fate programming. Computational identity scoring quantifies transcriptional similarity relative to reference atlases and enables cross-protocol comparison. Platform-Agnostic CellNet generates sample-level fidelity scores robust across platforms (Lo et al., 2023), while Cepo ranks genes by differential stability to derive per cell type assessments of transcriptional fidelity (Kim et al., 2023).

Complementary digital cytometry and deconvolution approaches, such as CIBERSORTx (Newman et al., 2019) and EPIC (Racle and Gfeller, 2020), infer cell type proportions and, in some cases, cell-type-specific expression profiles from bulk transcriptomes using reference signatures. These approaches provide indicators of compositional fidelity, particularly when single-cell data are unavailable or when evaluating heterogeneous differentiation outcomes and complex organoids.

However, transcriptional resemblance alone does not guarantee regulatory coherence or functional maturity. Quantitative identity scoring provides a useful but not sufficient criterion for evaluating engineered cell states.

### Computational benchmarking and prospective validation

Identity similarity alone does not establish whether a model captures the regulatory structure that governs fate transitions. Computational benchmarking therefore evaluates performance on specific tasks, including GRN reconstruction, enhancer-gene linking, trajectory inference, and lineage recovery.

For GRN inference, methods are typically assessed by recovery of known TF-target interactions using AUROC or AUPRC against ChIP-seq, curated regulons, or perturbation-derived interactions. Standardized resources such as BEELINE enable controlled comparisons under varying sparsity and heterogeneity (Pratapa et al., 2020). Enhancer-gene predictions can be benchmarked against perturbation assays such as CRISPRi-FlowFISH, with precision-recall and calibration analyses used to evaluate ranking accuracy and effect-size consistency (Fulco et al., 2019). Trajectory methods are evaluated by concordance with capture time, branch topology, or known lineage structure, as summarized in dynverse (Saelens et al., 2019), while lineage reconstruction tools quantify agreement with ground-truth clonal trees using metrics such as Robinson-Foulds distance (Jones et al., 2020).

Retrospective agreement with training datasets is insufficient to demonstrate predictive utility. For computational models to reduce experimental burden, they must reliably prioritize interventions such that testing a limited number of candidates yields reproducible gains in conversion efficiency, fidelity, or functional performance. This requires evaluation on held-out perturbations, time points, lineage contexts, or independently generated datasets that were not used during model development. Community-level evaluation initiatives, including the virtual cell challenge (Roohani et al., 2025), provide structured settings in which models are assessed on their ability to generalize to unseen conditions and reproduce predictions across laboratories. By emphasizing out-of-distribution testing and reproducibility, such frameworks move benchmarking beyond descriptive concordance toward prospective validation.

### Reference datasets, domain shift, and reproducibility

The validity of computational blueprints depends fundamentally on the quality and relevance of reference datasets. Cross-tissue atlases such as Tabula Sapiens (The Tabula Sapiens Consortium, 2022) provide harmonized adult profiles for annotation and identity scoring, while fetal atlases supply progenitor and transitional cells and states that are essential for modeling developmental trajectories (Cao et al., 2020). Regulatory and perturbational benchmarks likewise require lineage-appropriate data. Paired multi-omic datasets and curated TF-DNA interaction resources support benchmarking of enhancer-gene links and GRN edges, and perturbation compendia with single-cell readouts provide more stringent substrates for assessing causal predictions.

Mismatches in species, developmental stage, culture conditions, or assay chemistry can introduce domain shift that degrades predictive performance. To mitigate domain shift, studies should explicitly report reference selection criteria, harmonization workflows (e.g., batch correction or label transfer), and sensitivity analyses across alternative references. Predictive models should be evaluated on held-out tissues, laboratories, or perturbations to quantify transferability. For deep-learning approaches, reproducibility further requires release of training data provenance, model weights, inference pipelines, and version-controlled code.

Without careful matching of reference data and transparent reporting of evaluation procedures, studies may overestimate true predictive robustness. Establishing reproducible, lineage-specific reference resources will therefore be central to advancing computational cell fate programming beyond descriptive alignment toward reliable intervention design.

### Experimental validation and safety assessment

While computational metrics provide evidence of transcriptional fidelity and regulatory plausibility, experimental validation provides the decisive test of engineered cell fates, establishing functional competence, mechanistic correctness, and safety beyond transcriptomic similarity.

For identity validation, immunostaining and flow cytometry against predicted marker panels can be paired with chromatin-level assays such as scATAC-seq or targeted TF binding analyses. Functional assays must be lineage-specific and benchmarked against primary *in vivo* counterparts. Neurons and cardiomyocytes can be assessed using electrophysiology or calcium imaging (Karakikes et al., 2015); cardiomyocytes additionally through contractility and action-potential profiling (Lan et al., 2013); hepatocytes via metabolic function assays (Si-Tayeb et al., 2010); and beta cells through glucose-stimulated insulin secretion (Pagliuca et al., 2014). Functional convergence with primary tissues provides stronger evidence of successful programming than transcriptomic resemblance alone.

Mechanistic validation tests whether predicted regulators or enhancers exert the expected effects. Targeted perturbations using CRISPRa-i (Gilbert et al., 2013), enhancer perturbation assays such as CRISPRi-FlowFISH or MPRA (Fulco et al., 2019; Inoue and Ahituv, 2015), and timed intervention experiments derived from trajectory models (Bergen et al., 2020) enable causal interrogation of computational predictions. Lineage barcoding can independently confirm predicted fate biases (Weinreb et al., 2020).

For translational applications, safety assessment should include karyotype stability, off-target editing analysis, long-term phenotype persistence, and, where appropriate, *in vivo* engraftment or tumorigenicity testing. Rigorous practice requires biological replication, dose-response and time-course analyses, and transparent reporting of reagents and culture conditions.

Computational models meaningfully reduce experimental burden only when predicted gains in fidelity are reproducibly matched by functional and mechanistic recovery. Establishing concordance across molecular, functional, and safety dimensions is therefore essential for translating computational blueprints into reliable protocols for cell fate programming.

## Toward a design-test-learn pipeline for cell fate programming

The methods reviewed in foundations of computational cell fate modeling; the single-cell omics revolution; from identification to modulation; advances in deep learning for cell fate programming; and computational evaluation and experimental validation of engineered cell fates address complementary aspects of cell fate programming. Their practical value increases when integrated into a structured design-test-learn pipeline that explicitly alternates between computational modeling and experimental perturbation and validation.

1. Model-based nomination: single-cell foundation models and GRN inference identify candidate regulators, signaling pathways, or regulatory elements associated with a target cell type/state.
2. Experimental perturbation: selected candidates are implemented experimentally using TF overexpression, CRISPRa-i, small molecule treatment, or enhancer perturbation. Single-cell readouts (e.g., scRNA-seq, multiome profiling, etc) quantify how these interventions reshape transcriptional programmes, trajectories, and lineage outcomes (i.e., intervention-outcome relationships).
3. Perturbation modeling: the resulting perturbation data are incorporated into perturbation-aware models to refine intervention-outcome mappings. The trained models are then used for generative prediction of responses to unseen perturbations, higher-order combinations, or temporal schedules.
4. Contextual refinement: sequence-to-function and spatiotemporal models provide orthogonal constraints, informing regulatory element selection, timing, and spatial delivery of cues. These are used for model refinement and prediction.
5. Evaluation and decision point: engineered outcomes are benchmarked using quantitative fidelity metrics, held-out validation criteria, and functional assays (computational evaluation and experimental validation of engineered cell fates). If predefined performance thresholds (e.g., fidelity, efficiency, and functional maturity) are not met, the process returns to step 1 with updated priors. If criteria are satisfied, protocols proceed to extended functional and safety validation.

**Figure 4:**
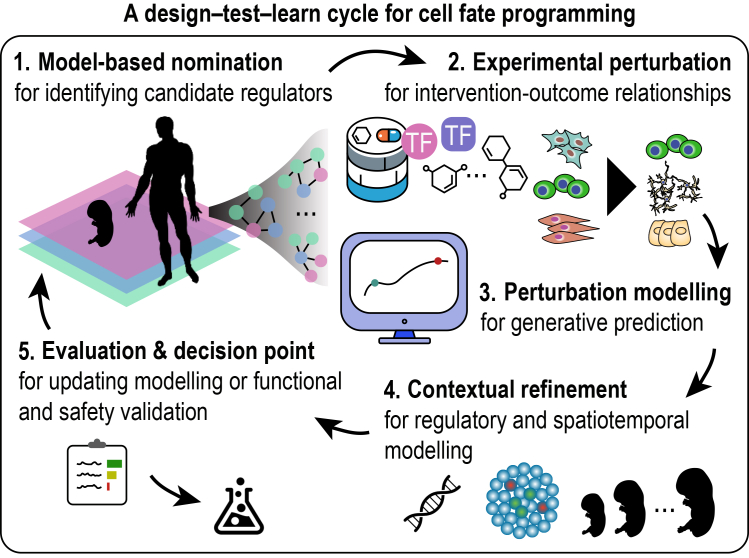
Design-test-learn cycle for cell fate programming Conceptual overview of an integrated computational-experimental workflow linking regulatory inference to functional validation. Candidate regulators are first nominated using single-cell and network-based models, then experimentally perturbed and profiled to establish intervention-outcome relationships. Perturbation-aware and generative models are trained to predict responses to new combinations or temporal schedules, while sequence-level and spatiotemporal constraints refine regulatory targeting and delivery strategies. Engineered states are quantitatively benchmarked for fidelity and function, and outcomes inform iterative model updating or progression to extended validation.

In this framework (Figure 4), experiments occur primarily at the perturbation stage and at the evaluation stage, and the loop is closed through systematic incorporation of experimentally measured effects into subsequent modeling rounds. The objective is not to eliminate empirical optimization, but to progressively reduce combinatorial search space and increase reproducibility through iterative refinement.

## Challenges and future directions

Despite substantial progress in computational methodologies for modeling and programming cell fate, several challenges remain before these approaches can be broadly applied in translational settings. Here, I focus on the computational perspective and highlight key obstacles and emerging directions.

### Data quality, standardization, and harmonization

The reliability of computational blueprints depends critically on the quality and consistency of underlying datasets. Early microarray and RNA-seq studies were often limited by substantial batch effects, heterogeneous sequencing depth, incomplete metadata annotation, and variable reporting standards, complicating cross-study integration and reducing confidence in inferred regulatory relationships. The improvements in library preparation, sequencing chemistry, quality control, and centralization (e.g., core facilities, commercial providers, etc.) have markedly enhanced data comparability. These advances have strengthened cross-study integration and model training. Nevertheless, variation in culture conditions, developmental context, and lineage specification protocols continues to introduce context-dependent biases, underscoring the need for rigorous harmonization and integration frameworks.

### Causal inference and regulatory complexity

Reconstructing molecular networks that capture causal relationships across diverse contexts remains a major challenge. Existing models can perform well in narrow settings but often fail to generalize to new cell types or perturbations. Progress will require systematic perturbation atlases, improved combinatorial intervention designs, and causal frameworks that extend beyond transcriptional regulation to incorporate signaling pathways and cell-cell communication within organoids and tissue microenvironments.

### Spatiotemporal data integration

While spatiotemporal datasets are increasingly available, integrating temporal trajectories with three-dimensional spatial organization remains computationally demanding. Combining lineage tracing, live-cell imaging, and spatial multi-omics into coherent modeling frameworks will be essential for understanding how extracellular cues and tissue architecture shape cell-fate decisions. Such integration moves beyond static molecular predictions toward context-aware design of tissues and organoids.

### Deep learning, foundation models, and generalization

Large-scale foundation models trained on millions of single-cell profiles provide new opportunities for modeling cell identity and fate. However, training datasets often overrepresent well-studied tissues and systems, raising concerns about systematic bias and limited transferability to under-characterized contexts. Improving uncertainty quantification and establishing prospective validation frameworks will be essential to ensure that such models offer experimentally actionable guidance rather than retrospective alignment.

### From regulatory blueprints to virtual cells

The concept of computational blueprints intersects with earlier whole-cell modeling efforts and emerging “virtual cell” initiatives (Roohani et al., 2025). Classical whole-cell modeling sought comprehensive mechanistic integration across metabolism, gene regulation, and signaling networks, whereas more recent virtual cell frameworks leverage large-scale single-cell data and generative modeling to forecast cellular responses to perturbations. The blueprint framework proposed here is narrower in scope. Rather than attempting full mechanistic simulation, it focuses on predictive mapping between defined interventions and lineage-specific fate outcomes, grounded in experimentally measured perturbation data and iterative validation. Achieving fully generalizable virtual cell systems will require expanded perturbational coverage, improved cross-context generalization, and continued advances in data quality and evaluation frameworks.

### Reproducibility, governance, and responsible innovation

Ensuring reproducibility across computational predictions and experimental workflows remains a persistent challenge. Transparent reporting, open sharing of computational models and training data provenance, and rigorous documentation of experimental protocols are essential for independent verification. Establishing community benchmarks and quantitative fidelity frameworks will further support cross-laboratory validation. Ethical considerations must accompany computational cell fate programming; for example, computer-guided differentiation of stem cells into embryo-like structures such as blastoids raises important questions regarding safety and oversight. Responsible innovation requires engagement with regulators, ethicists, and patient communities to ensure safe and equitable translation.

Taken together, these challenges indicate that future progress will depend not only on algorithmic advances but also on sustained improvements in data infrastructure, perturbation resources, evaluation standards, and translational governance. Continued development of structured design-test-learn pipelines that couple computational prediction with prospective validation offers a pathway toward more reliable and reproducible cell fate programming.

## Conclusions

Computational models have evolved from early dynamical systems to multimodal single-cell and spatial omics frameworks increasingly supported by deep learning. These advances have improved our ability to characterize cell states, infer regulatory structure, and prioritize candidate interventions. Rather than replacing empirical optimization, computational blueprints provide structured approaches to narrow combinatorial search space within iterative design-test-learn cycles. Realizing predictive and reproducible cell fate programming will require high-quality perturbation data, rigorous benchmarking, transparent evaluation, and orthogonal functional validation. With continued integration of computation and experimentation, computational blueprints will progressively improve the design of more efficient and reproducible cell fate programming protocols, advancing disease modeling, drug discovery, tissue engineering, regenerative medicine, and bioproduction.

## Acknowledgments

P.Y. is supported by an 10.13039/501100000923Australian Research Council (ARC) Future Fellowship (FT250100252). P.Y. thanks Professor Patrick Tam and members of the Computational Systems Biology Unit for their valuable feedback on the manuscript. A large language model (ChatGPT-5, OpenAI) was used to assist with copy-editing during manuscript preparation.

## Author contributions

P.Y. conceptualized this work, reviewed the literature, wrote the manuscript, and approved the final manuscript.

## Declaration of interests

P.Y. is an early career editor of Stem Cell Reports.
